# Complete genome sequence of the *Escherichia coli* phage vB_Ec_Tarrare

**DOI:** 10.1128/MRA.00828-23

**Published:** 2023-10-31

**Authors:** Forrest I. Veilleux, Ibrahim Ayyash, Shallee T. Page

**Affiliations:** 1 College of Health and Natural Sciences, Franklin Pierce University, Rindge, New Hampshire, USA; Portland State University, Portland, Oregon, USA

**Keywords:** phage, bacteriophage, genome analysis, genomics, Teseptimavirus

## Abstract

We report the isolation, sequencing, and annotation of the novel bacteriophage vB_Ec_Tarrare, which infects the *Escherichia coli* K-12 strain. It was isolated from a bat guano sample collected in Rindge, NH, USA. Its genome is 40,953 base pairs long with 49 putative protein-coding genes and no transfer RNAs.

## ANNOUNCEMENT

Both the coliform bacteria of bats and their bacteriophages are under-explored (1). *Escherichia coli* is responsible for an estimated 2,801,000 illnesses per year, resulting in 230 deaths (2). Exploration of phages specific to coliform bacteria with pathogenic strains may expand treatment options for bacterial gastroenteritis (3).

Here, we describe vB_Ec_Tarrare, a 40,953 bp double-stranded DNA bacteriophage isolated from an *Eptesicus fuscus* guano on 6/20/22 at coordinates 42.74421N 72.01907W. vB_Ec_Tarrare was isolated through co-culture of 10 pellets of bat guano with 500 µL *E. coli* K-12 HB101 in 30 mL LB broth at 37°C for 24 hours. The resulting crude phage enrichment was filter-sterilized and then reinoculated with *E. coli* K-12 and plated on LB top agar at 37°C. This sample was purified with three rounds of serial dilution and plaque isolation (clear plaques about 0.5 cm in diameter). A high-titer lysate was prepared from the purified phage by flooding webbed plaque plates with buffer (20 mM Tris-HCl, 100 mM NaCl, 10 mM MgSO_4_, 0.1 M CaCl_2_) and filter-sterilizing the lysate (4). Bacterial contaminants were removed with DNAse, RNAse, and proteinase K (Sigma). Viral DNA was extracted with the Zymo-Spin Viral Kit (#D3015), quantitated *via* NanoDrop (ThermoFisher), and assessed for quality with gel electrophoresis.

The DNA was assessed using ScreenTape (Agilent), prepared using the TruSeq DNA Nano library prep kit (Illumina) *via* random fragmentation, and subjected to bead-based size selection followed by ligation of dual-index adapters in multiplex runs. Paired-end sequencing (Illumina NovaSeq6000) yielded 2.73 × 10^6^ reads of an average length of 145 nucleotides (nt) and 323-fold coverage of the genome.

The software was run using default settings except where otherwise noted. Assessment of read quality, adaptor trimming, assembly, and determination of termini was done using CLC Genomics workbench v.21 assembly tool v.6.5.1 (Qiagen) at NCSUGSL. The 40,953 bp genome has a G/C percentage of 47.3%. Genome auto-annotation of the complete genome was performed at Franklin Pierce University using DNAMaster v5.23.6 (http://cobamide2.bio.pitt.edu), Glimmer v3.02b (5), and GeneMark v3.25 (6). Translational start sites were manually confirmed using coding potential prediction (7) from GeneMark.hmm v2.5 (http://exon.gatech.edu/GeneMark/heuristic_gmhmmp.cgi), synteny with related phages, overlap with nearby genes, and ribosomal binding site scores from DNAMaster. Putative gene functions were assigned with NCBI BlastP (8) and HHPred (9).

**Fig 1:**
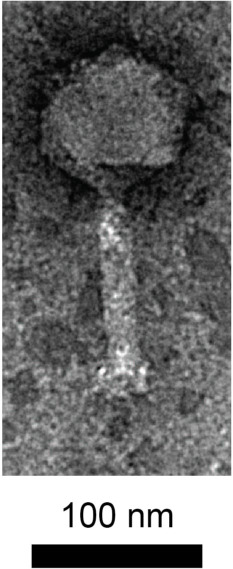
Transmission electron micrograph of *Escherichia coli* phage vB_Ec_Tarrare captured on a FEI Tecnai F20 TEM operated at 200KV. Scale bar: 100 nm.

Nucleotide similarity *via* BLASTn (8) (nr; stringency: discontinuous megablast) predicted that vB_Ec_Tarrare is a Teseptimavirus. Its closest relative is the *Escherichia* phage pO111 (genome blastn yields 75% nucleotide identity, 76% coverage). BLASTX alignment (nr database) (8) of the well-conserved DNA-directed RNA polymerase shows ~88% identity with a range of phages including Shigella phage Esh3. Notable predicted features of the genome include a Type II holin and a suppressor of silencing. Transmission electron microscopy of a sample negative-stained with 1% uranyl acetate revealed a capsid of ~90 nm and a tail length of ~120 nm (Fig. 1).

## Data Availability

Data for vB_Ec_Tarrare deposited by S. Page, Franklin Pierce University: Bioproject: 1008589, Biosample SAMN34534539, Short Read Archive SRX21470829, NCBI Taxonomy Browser: 3032379, NCBI NCBI Nucleotide: OQ507919.
